# Spectrum of heart diseases in a new cardiac service in Nigeria: An echocardiographic study of 1441 subjects in Abeokuta

**DOI:** 10.1186/1756-0500-1-98

**Published:** 2008-10-28

**Authors:** Okechukwu S Ogah, Gail D Adegbite, Rufus O Akinyemi, Julius O Adesina, Albert A Alabi, Oscar I Udofia, Roseline F Ogundipe, Julius KL Osinfade

**Affiliations:** 1 Department of Medicine, Federal medical centre, Idi-Aba, Abeokuta, Ogun State, Nigeria; 2 Department of Medicine, Sacred Heart Hospital Lantoro, Abeokuta, Ogun State, Nigeria

## Abstract

**Background:**

Echocardiography is a non-invasive, relatively cheap and useful imaging technique for the evaluation of cardiac diseases. The procedure has reliable levels of accuracy.

Echocardiography commenced at the Federal medical centre Abeokuta on September 9, 2005.

The aim of this study is to report our experience with the procedure, and to define the clinical cases seen in our setting.

**Methods:**

This is a retrospective analysis of a prospectively collected data. Echocardiography was performed using Aloka SSD 1,100 echocardiograph equipped with 2.5–5.0 MHz transducer

**Results:**

During the period of 18 months under review (September 2005–February 2007), 1629 procedures were performed. The reports of 188 echocardiograms were excluded due to poor echo-window, repeated procedure or incomplete report. 1441 reports were reviewed for demographic parameter, indications for the procedure and the main echocardiographic diagnoses.

The mean age of the 1441 individuals studied was 54 +/- 14.3 years (15–90). There were 744 men and 697 women. Eight hundred and seventeen subjects (56.7%) had hypertensive heart disease, 53 subjects (3.7%) had rheumatic heart disease while 44(3.0%) had dilated cardiomyopathy. Pericardial diseases, cor-pulmonale, ischaemic heart disease, congenital heart diseases, diabetic heart disease, thyroid heart disease, sickle cell cardiopathy were present in 26(1.8%), 23(1.6%), 9(0.6%), 6(0.4%), 6(0.4%), 6(0.4%), 1(0.1%), and 1(0.1%) respectively. Four hundred and forty nine (31.2%) subjects had normal study.

**Conclusion:**

Hypertensive heart disease was found to be the most prevalent cardiac condition in this study. The relatively frequent diagnoses of rheumatic heart disease, cardiomyopathies and pericardial diseases reflect the impact of infections and infestations on the cardiovascular health of adult Nigerians.

We suggest that prevention and treatment of cardiac diseases in our setting should among other things focus on blood pressure control and early treatment of infections causing heart diseases.

## Background

Epidemiological studies have shown that heart diseases are on the increase worldwide especially in low income countries and developing economies where non-communicable diseases are emerging [1]

Cardiovascular disease now constitute major heath problem in developing nations. Over 80% of global morbidity and mortality from cardiovascular diseases now occur in these countries.

Knowledge of the prevalent and patterns of heart diseases in any environment is important in health care planning and in the provision of health care services.

Echocardiography has grown to become the most widely used cardiac imaging technique worldwide. In 2003, the medical world marked the 50^th ^anniversary of the original demonstration of cardiac ultrasound by Edler and Hertz.

Although the procedure had an unheralded beginning, it has become a powerful tool in the diagnosis of cardiac diseases and in epidemiological studies.

In 2003, it was estimated that over 20 million procedures were performed in the United States of America alone [2].

At the Federal Medical Centre Abeokuta, Nigeria, M-mode and 2-dimentional echocardiography commenced on the 9^th ^day of September 2005.

The aim of this study is to report on our experience with the procedure, and to describe the different clinical cases diagnosed with this tool over a period of 18-months.

## Methods

This is retrospective and descriptive study of a prospectively collected data. The study was carried out at the Department of Medicine, Federal Medical Centre, Abeokuta, Nigeria between September 2005 and February 2007.

The centre is a relatively young tertiary one, established in 1993 by the Federal Government of Nigeria to cater for the health need of the people of Ogun State and its environs in Southwestern Nigeria. The state has a population of about 3.2 million and a land area of about 16,409.26 square kilometers.

Echocardiography is performed at our centre on a twice weekly basis except in emergency situations.

Ethical approval was obtained from our institution's ethical review committee.

### Clinical Evaluation

Baseline clinical and demographic characteristics were obtained from the subjects. These included: date of birth, age, gender and indication for echocardiogram.

### Echocardiography

Two-dimensional guided M-mode echocardiography with the use of commercially available echo-machine (ALOKA SSD-1, 100) and a 2.5–5.0 MHz linear array transducer was performed on each subject in the partial decubitus position. All measurements were made according to the American Society of Echocardiography (ASE) leading edge to leading edge convention [3]. Echocardiographic examination was performed in the parasternal long axis, short axis, apical four chamber and occasionally in the subcostal and suprasternal views. LV measurement was obtained at end diastole and end systole in the parasternal long axis view. The LV measurements taken include right ventricular outflow tract diameter (RVOT), aortic root diameter (AO), and aortic valve opening (AVO) and left atrial diameter (LA). Others include interventricular septal thickness at end-diastole (IVSTd) and end-systole (IVSTs), the posterior wall thickness at end diastole (PWTd) and end-systole (PWTs), and the LV internal dimensions at end systole (LVIDs) and end diastole (LVIDd). The end of diastole was taken as the peak of the R-wave of the ECG tracing on the echocardiograph while the end-systolic measurements were taken at the nadir of the LV septal wall [3].

One experienced cardiologist performed all the echocardiography. In our laboratory, the intra-observer concordance correlation coefficient and measurement error have been reported. [4].

All the echocardiographic diagnoses were based on standard criteria.

Hypertensive heart disease was diagnosed in the presence of any or combination of the following abnormalities: left ventricular systolic dysfunction (ejection fraction < 50%), left ventricular hypertrophy (indexed LV mass > 51 g/m^2.7^), and dilated left atrium, a surrogate of impaired LV filling (left atrial diameter > 3.8 cm in women and > 4.2 cm in men).

Left ventricular geometric patterns were defined according to Ganau et al [5].

Valvular heart diseases (mostly rheumatic in origin) were documented based on the following:

i. Mitral stenosis: – presence of thickened and calcified mitral valve leaflets, loss of the classic M-shaped pattern of a normal mitral valve, diastolic dooming and restriction of the mitral valve leaflet motions.

ii. Mitral Regurgitation: Poor coaptation of the mitral valve leaflets in systole, thickened leaflets, dilated and hyperdynamic left ventricle

iii. Aortic stenosis: Presence of calcified aortic valve, reduction in aortic cusp separation, highly echo reflectant aortic valve leaflets

iv. Aortic regurgitation: Poor coaptation of the aortic cusps in diastole dilated left ventricles and fine fluttering of the anterior mitral valve in diastole.

Dilated cardiomyopathy was diagnosed when there are dilated heart chambers with normal or decreased wall chambers as well as impaired LV systolic function [6].

Endomyocardial fibrosis (EMF) was documented in the presence of clinical features coupled with dilated atria and thickening of the endocardium especially at the apices of the ventricles [6].

Pericardial effusion was diagnosed when there is echo free space between the visceral and parietal pericardium. Cor-pulmonale was present when there is dilated and hypertrophied right ventricle (RV), evidence of increased RV systolic pressure (D-shaped LV in diastole (diastolic flattening of the LV septum)

### Data Analysis

Data management and analysis were performed with SPSS software version 11.0. (SPSS, Inc. Chicago, Illinois). Continuous variables were expressed as mean ± SD (standard deviation) and categorical variables expressed as percentages. Differences in categorical variables were assessed by Chi-square analysis.

A 2-tailed p value < 0.05 was considered to be significant.

## Results

During the 18 months period 1,629 echocardiograms were performed. The data of 188 subjects were excluded in the analysis due to poor echo-window, repeated procedure in the course of the management of the patient, or incomplete data.

One thousand, four hundred and forty one (1441) subjects were analyzed.

Table 1 shows the clinical and demographic characteristics of the subjects. There were 744 men and 697 women aged 55.4 ± 14.1 and 53.4 ± 14.3 years respectively.

**Table 1:** Demographic and clinical characteristics of the 1441 subjects.

**Parameter**	**All**	**Men**	**Women**
Number	1441	744	697
Age	54.4(14.3)	55.4(14.1)	53.4(14.3)
Age group			
<= 19	17(1.2)	8(1.1)	9(1.3)
20–29	43(3.0)	24(3.2)	19(2.7)
30–39	148(10.3)	68(9.1)	80(11.6)
40–49	291(20.2)	138(18.4)	153(22.1)
50–59	408(28.3)	213(29.1)	195(27.5)
60–69	278(19.3)	145(19.4)	133(19.2)
70–79	204(14.2)	115(15.4)	89(12.9)
>= 80	52(3.6)	33(4.4)	19(2.7)
Weight(kg)	71.9(14.3)	72.0(15.9)	71.8(28.3)
Height(cm)	164.1(9.2)	168.8(68.7)	159.3(55.2)*
BMI(kg/m^2^)	26.6(6.2)	25.1(5.1)	28.0(6.7)*
BSA(m^2^)	1.74(0.21)	1.82(0.20)	1.73(6.2)*
Pulse (beat/min)	85.2(14.4)	84.4(13.3)	86.0(15.4)*
Systolic BP(mmHg)	138.6(25.3)	137.7(24.6)	139.6(26.0)
Diastolic BP(mmHg)	85.9(15.6)	85.9(16.4)	86.0(14.6)

BMI = body mass index, BSA = body surface area, BP = blood pressure. * indicates P < 0.05

The mean age of all the subjects was 54.4 ± 14.3 years and range 15–90 years.

Table 2 shows the indications for referral for echocardiography. More than 60% were because of hypertension or hypertensive heart disease. Other reasons for referral include congestive heart failure (12.1%), valvular heart disease (3.4%), dilated cardiomyopathy (2.6%), stroke/TIA (2.4%) and pericardial disease (1.3%).

**Table 2:** Indications for Echocardiography in the 1441 subjects.

**SNO**	**Indication**	**Frequency**	**Percent (%)**
1	Abnormal ECG	9	0.6
2	Cardiac murmur	8	0.6
3	Cardiomegaly on CXR	1	0.1
4	Congestive Heart Failure	175	12.1
5	Complete Heart Block	2	0.1
6	Congenital Heart Disease	5	0.3
7	Cor-pulmonale/Pulmonary Hypertension	37	2.6
8	Dilated cardiomyopathy	37	2.6
9	Diabetes Heart Disease	21	1.4
10	Hypertension/Hypertensive Heart Disease	944	65.5
11	Ischaemic Heart Disease	12	0.9
12	Myocarditis	1	0.1
13	Palpitation	18	1.3
14	Pericardial Diseases	19	1.3
15	Pre-chemotherapy	3	0.2
16	Pre-operative cardiac evaluation	7	0.5
17	Restrictive Heart Disease	3	0.2
18	Routine medical check-up	9	0.6
19	Stroke/TIA	35	2.4
20	Thyroid Heart Disease	12	0.9
21	Unexplained Dyspnoea	12	0.9
22	Unexplained leg swelling	2	0.1
23	Valvular Heart Disease	50	3.4
24	Not specified	19	1.3
	**Total**	**1441**	**100.0**

Eight hundred and seventeen subjects (56.7%) had hypertensive heart disease, 53 subjects (3.7%) had rheumatic heart disease while 44(3.0%) had dilated cardiomyopathy. Pericardial diseases, Cor-pulmonale, ischaemic heart disease, congenital heart diseases, diabetic heart disease, thyroid heart disease, sickle cell cardiopathy were present in 26(1.8%), 23(1.6%), 9(0.6%), 6(0.4%), 6(0.4%), 6(0.4%), 1(0.1%), and 1(0.1%) respectively. Four hundred and forty nine (31.2%) subjects had normal study (Table 3).

**Table 3:** Echocardiographic Diagnoses in the 1441 subjects

	**All (n = 1441)**		**Males(n = 744)**		**Females (n = 697)**	
**Diagnosis**	**Frequency**	**Percent**	**Frequency**	**Percent**	**Frequency**	**Percent**
**Hypertensive Heart Disease**	**817**	**56.7**	**447**	**60.2**	**370**	**53.0**
**Normal Study**	**449**	**31.2**	**207**	**27.8**	**242**	**34.6**
**Valvular Heart Disease**	**53**	**3.7**	**20**	**2.8**	**33**	**4.7**
• Rheumatic MR	30	-	12	-	18	-
• Rheumatic MS	8	-	4	-	4	-
• Rheumatic AR	2	-	2	-	0	-
• Mixed type	7	-	4	-	3	-
• Degenerative MS	1	-	0	-	1	-
• AR Associated with Marfan's Syndrome	1	-	1	-	0	-
• AS associated with Bicuspid aorta	1	-		-	0	-
• Mitral Valve Prolapse	3	-	3	-	0	-
**Cardiomyopathy**	**44**	**3.0**	**21**	**2.8**	**23**	**3.3**
• Idiopathic DCM	34	-	18	-	16	-
• Doxorubicin Induced CM	1	-	0	-	1	-
• Peripartum CM	4	-	0	-	4	-
• EMF	4	-	2	-	2	-
• HOCM	1	-	1	-	0	-
**Pericardial Diseases**	**26**	**1.8**	**20**	**2.8**	**6**	**0.9**
Pericarditis	25	-	19	-	6	-
• Effusive	8	-	4	-	4	-
• Effusive-constrictive	13	-	12	-	1	-
• Constrictive	4	-	3	-	1	-
Pericardial cyst	1	-	1	-	0	-
**Cor-pulmonale**	**23**	**1.6**	**13**	**1.8**	**10**	**1.4**
• COPD	20	-	12	-	8	-
• HIV Associated Pulmonary Hypertension	2	-	1	-	1	-
• Primary Pulmonary Hypertension	1	-	0	-	1	-
**Ischaemic Heart Disease**	**9**	**0.6**	**8**	**1.1**	**1**	**0.1**
**Adult Congenital Heart Disease**	**6**	**0.4**	**1**	**0.1**	**5**	**0.7**
• Ostium primum ASD	1	-	0	-	1	-
• Dextrocardia	1	-	0	-	1	-
• VSD	1	-	1	-	0	-
• Pulmonary stenosis	1	-	0	-	1	-
• Tetralogy of Fallot	1	-	0	-	1	-
• Partial endocardial cushion defect	1	-	0	-	1	-
**Diabetic Heart Disease**	**6**	**0.4**	**4**	**0.5**	**2**	**0.3**
**Thyroid Heart Disease**	**6**	**0.4**	**0**	**0.00**	**6**	**0.9**
**Sickle Cell Cardiopathy**	**1**	**0.1**	**0**	**0.00**	**1**	**0.14**
**Atrial Tumour**	**1**	**0.1**	**1**	**0.1**	**0**	**0.00**

The commonest type of valvular heart disease was pure rheumatic mitral regurgitation (30 cases). This is followed by pure mitral stenosis (8 cases), and 7 cases of mixed rheumatic heart disease. Three cases of primary mitral valve prolapse were documented.

The commonest form of pericardial disease was effusive-constrictive pericarditis (13 cases) followed by effusive type (8 cases) and constrictive pericarditis (4 cases)

Idiopathic dilated cardiomyopathy was the commonest form of cardiomyopathy (constituting 34 of the 44 cases seen)

There were 6 cases of adult congenital heart disease.

**Figure 1:**
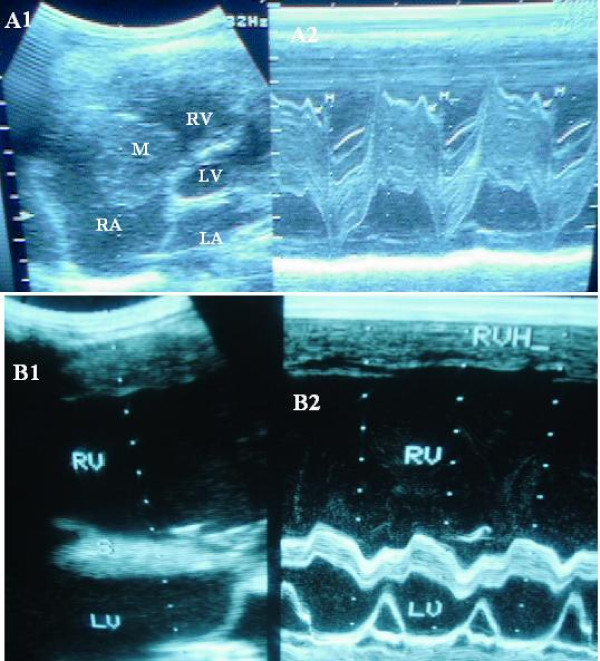
**A(1&2).** 2D and M-mode echocardiogram of a subject with right atrial tumour; B(1&2). 2D and M-mode echocardiogram of a subject with Cor-pulmonale.

**Figure 2:**
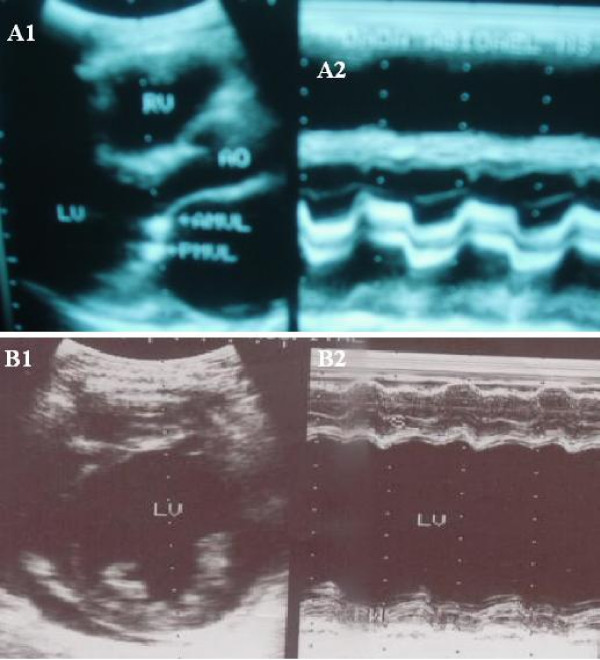
**A(1&2). **2D and M-mode echocardiogram of a subject with mitral stenosis; B(1&2). 2D and M-mode echocardiogram of a subject with idiopathic dilated cardiomyopathy.

**Figure 3:**
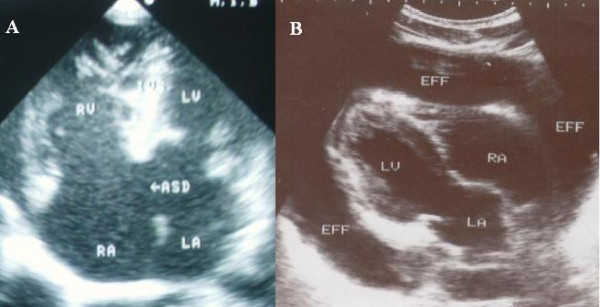
A. echocardiogram of a subject with a large atrial septal defect; B. 2D echocardiogram of a subject with effusive pericarditis.

Figures 1, 2, 3 are examples of the cases documented.

## Discussion

This echocardiographic based study shows that the common heart diseases in adult Nigerians in Abeokuta are hypertensive heart disease, valvular heart disease (mostly rheumatic), dilated cardiomyopathy, pericardial diseases, and cor-pulmonale. Ischaemic heart disease and adult congenital heart disease were infrequently diagnosed (Table 4).

**Table 4:** Spectrum of echocardiographic diagnosed heart diseases (excluding individuals with normal study)

	**All**		**Males**		**Females**	
**Diagnosis**	**Frequency**	**%**	**Frequency**	**%**	**Frequency**	%
Hypertensive Heart Disease	817	82.4	447	83.6	370	81.0
Valvular Heart Disease	53	5.3	20	3.7	33	7.2
Cardiomyopathy	44	4.5	21	3.9	23	5.0
Pericardial Diseases	26	2.6	20	3.7	6	1.3
Cor-pulmonale	23	2.3	13	2.4	10	2.3
Ischaemic Heart Disease	9	0.9	8	1.5	1	0.2
Congenital Heart Disease	6	0.6	1	0.2	5	1.1
Diabetic Heart Disease	6	0.6	4	0.8	2	0.4
Thyroid Heart Disease	6	0.6	0	0.0	6	1.3
Atrial Tumour	1	0.1	1	0.2	0	0.0
Sickle Cell Cardiopathy	1	0.1	0	0.0	1	0.2
TOTAL	992	100	535	100	457	100

Hypertensive heart disease is by far the commonest heart disease in this study. It is well established that this condition forms the bulk and is the foundation of cardiovascular disease in Africa.

The relatively frequent diagnoses of rheumatic heart disease, cardiomyopathies and pericardial diseases reflect the impact of infections and infestations on the cardiovascular health of adult Nigerians.

The large number of normal findings at echocardiography in this study is similar to some previous studies in Nigeria [7]. This can be explained by the fact that many of the referrals came from all cadres of physicians and most often the subjects were not properly screened for heart diseases before referral. Moreso many hypertensive patients with abnormal electrocardiogram were referred for cardiac function evaluation. The poor predictive value of electrocardiography in identifying patients with cardiac abnormality has been well established.

Our findings are similar to previous similar studies from other parts of Nigeria.(Table 5)

**Table 5:** Comparison of present study with previous studies in Nigeria

Parameter	Present study	Aje et al[20]	Sani et al [7]*	Ike et al[21]	Ukoh et al[22]*	Agomuoh et al[23]	Balogun et al[9]	Adesanya et al[8]*
Number	1441	1544	594	2527	869	141	100	249
Duration	18 mths	19 mths	24 mths	10 yrs	8 yrs	36 mths	30 mths	12 mths
HHD	817	687	228	436	249	48	53	24
VHD	53	54	64	868	152	13	7	14
CM	44	40	144	237	165	28	21	14
PD	26	22	7	228	12	6	4	20
CP	26	15	7	31	12	1	0	0
IHD	9	18	23	20	22	0	2	0
CHD	6	10	6	334	52	2	0	0
NS	449	674	100	275	58	43	13	17

* Classification of various conditions was reclassified. Only the main common diagnoses were included. HHD = Hypertensive Heart Disease, VHD = Valvular Heart Disease, CM = Cardiomyopathy, PD = Pericardial Diseases, CP = Cor-pulmonale, IHD = Ischaemic Heart Disease, CHD = Congenital Heart Disease, NS = Normal Study

In a review of 275 echocardiograms performed in Zaria over a 12-month period (1976–1977), Adesanya et al [8] reported similar findings. In a preliminary audit of 100 two-dimensional and Doppler echocardiographic service in a tertiary private hospital in the country, Balogun et al[9] found that the echocardiographic diagnosis of the aetiology of heart diseases are as follows: hypertensive heart disease (53%), cardiomyopathies (21%), valvular heart disease (7%), pericardial effusion (4%) and ischaemic heart disease (2%). Thirteen percent of their procedure was reported as normal.

Ukoh et al [10] in Benin reviewed 869 patients referred for echocardiography in Benin City (between January 1992 and May 2001), hypertensive heart disease (32.7%), dilated cardiomyopathy (19.2%), rheumatic heart disease (18.1%), and pericardial diseases (12.1%) were the common heart diseases identified. Ischaemic heart disease was uncommon (2.9%). Fifty eight studies were reported as normal.

Further analysis of the 817 hypertensive patients in this study shows that left ventricular hypertrophy (LVH) either concentric LVH or eccentric LVH was present in 53%. The frequency of LV geometry is as follows in the order of decreasing frequency: concentric LVH (40.2%), concentric remodeling (30.2%), normal LV geometry (16.8%) and eccentric LVH (12.8%).

About forty percent (39.6%) including 49.1% of women and 30.1% of men had dilated left atrium while about twenty percent (19.5%) had impaired LV systolic function.

The spectrum of chronic rheumatic heart disease in this study is similar to earlier finding in the country with mitral regurgitation being the commonest valve lesion.

We found dilated cardiomyopathy as the commonest form of cardiomyopathy in Abeokuta similar to other reports. However, peripartum cardiomyopathy (PPCM) is relatively uncommon compared to reports from the Northern part of the country. Endomyocardial fibrosis (EMF) was diagnosed infrequently in our study compared to reports in the 60s and 70s.

The low frequency of hypertrophic cardiomyopathy is similar to reports from the country. Our report also demonstrated the low prevalence of ischaemic heart disease in the country as reported by previous authors [11-13].

Echocardiography (even where M-mode and 2D alone are available) has been shown to be a useful tool in establishing cardiac diagnosis and in evaluating the performance of the heart in various disease conditions.

In resource poor setting like ours, it is ideal because it is non-invasive.

Worldwide the tool has been shown to be a very useful in clinical patient care and research.

In 2003 alone (when the world celebrated the 50^th ^year of existence of the tool), one can identify more papers using the search term "echocardiography "than in the first 25 years after Edler's initial description combined.

Echocardiography was introduced in Nigeria in the mid 70s mostly in the teaching hospitals. Its growth however has been very slow compared to advanced countries and some developing countries. Currently conventional M-mode, 2-dimensional echocardiography is mostly performed. Some institutions have Doppler and colour echocardiography. Hand-held or portable echocardiograph is probably available in only two centres. Transoesophageal and 3-dimensional echocardiography is not yet available in the country.

The potential of echocardiography as a research tool in Nigeria cannot be overemphasized. Studies emanating from the country have focused on the common cardiovascular diseases in the country such as hypertensive heart disease [14], heart failure, dilated cardiomyopathy including peripartum heart disease[15], and valvular heart disease. Others have also studied cardiac function in diabetes mellitus [16] chronic renal failure, congenital heart diseases[17], mitral valve prolapse[18], sickle cell disease and normal Nigerians. The usefulness of ECG criteria for the diagnosis of left ventricular hypertrophy in Nigerians using echocardiography as standard has also been reported [19].

## Conclusion

Our study shows that hypertensive heart disease, chronic rheumatic heart disease, cardiomyopathy, Cor-pulmonale and pericardial diseases are the common causes of heart disease in Abeokuta (South-West Nigeria).

It also shows the increasing burden of non-communicable diseases in the country coupled with the impact of infective conditions on the heart such as tuberculosis.

There is therefore need for strategies to control cardiovascular risk factors such as hypertension, obesity, and physical inactivity.

There is also need for improvement in housing and environmental sanitation as well early detection and treatment of throat infection

## Competing interests

The authors declare that they have no competing interests.

## Authors' contributions

OSO and GWA conceived the study. JOA, AAA, ROA, RFO and JKO participated in its design. OSO performed the echocardiographic examinations. OIU, JOA, and AAA participated in the recruitment of subjects and collated the data. OSO, OIU and GWA managed the data, performed statistical analysis. OSO drafted the manuscript. All authors read and approved the final manuscript.
